# Effect of enzyme supplements on macronutrient digestibility by healthy adult dogs

**DOI:** 10.1017/jns.2017.10

**Published:** 2017-04-18

**Authors:** Cecilia Villaverde, Edgar G. Manzanilla, Jenifer Molina, Jennifer A. Larsen

**Affiliations:** 1 Departament Ciencia Animal i del Aliments, Universitat Autònoma de Barcelona, Edifici V, Campus UAB, 08193 Bellaterra, Spain; 2 Pig Development Department, Animal and Grassland Research and Innovation Centre, TEAGASC, The Irish Food and Agriculture Authority, Moorepark, Fermoy, P61 C996, Co Cork, Republic of Ireland; 3 Department of Molecular Biosciences, School of Veterinary Medicine, University of California, Davis, One Shields Avenue, Davis, CA, USA

**Keywords:** Dogs, Digestive enzymes, Nutrient digestibility, Pancreatic enzyme supplementation, Plant-origin enzymes, AAFCO, Association of American Feed Control Officials, CP, crude protein, EE, ether extract, EPI, exocrine pancreatic insufficiency, GE, gross energy, TLI, trypsin-like immunoreactivity

## Abstract

Some enzyme supplement products claim benefits for healthy dogs to compensate for alleged suboptimal production of endogenous enzymes and the loss of enzymes in commercial pet foods secondary to processing. The objective of the current study was to determine macronutrient and energy digestibility by healthy adult dogs fed a commercial maintenance diet with or without supplementation with plant- and animal-origin enzyme products at the dosage recommended by their respective manufacturers. A group of fourteen healthy neutered adult Beagle dogs (average age 8 years) was divided into two equal groups and fed the basal diet alone and then with either the plant- or animal-origin enzyme supplement in three consecutive 10-d periods; the treatment groups received the opposite enzyme supplement in the third period. Digestibility in each period was performed by the total faecal collection method. Serum trypsin-like immunoreactivity (TLI) was measured at the end of each trial. Data were analysed by repeated measures and the α level of significance was set at 0·05. There were no differences in energy and nutrient digestibility between enzyme treatments. When comparing basal with enzyme supplementation, fat digestibility was higher for the basal diet compared with the animal-origin enzyme treatment, which could be a period effect and was not biologically significant (94·7 *v*. 93·5 %). Serum TLI was not affected by supplementation with either enzyme product. Exogenous enzyme supplementation did not significantly increase digestibility of a typical commercial dry diet in healthy adult dogs and routine use of such products is not recommended.

Digestive enzyme replacement therapy is an efficacious, evidence-based treatment for dogs and cats with exocrine pancreatic insufficiency (EPI)^(^^1^^)^. Most clinicians recommend only the use of animal-origin digestive enzyme replacement products that are mixtures of amylase, protease and lipase sourced from porcine pancreas; however, owners sometimes use plant-origin products as a substitute due to cost. In addition, the use of both animal- and plant-origin enzyme supplement products for all pets, including those without EPI, is advocated by some veterinarians and is a common practice among pet owners. A frequent claim is that this practice will compensate for the alleged suboptimal production of endogenous enzymes by the pancreas of healthy dogs as well as the loss of enzymes present in commercial pet foods secondary to processing and cooking. Several benefits are claimed by advocates of this practice, ranging from improvement in digestibility of nutrients to support of the immune system. Although some enzyme products of animal origin have been approved as drugs by the United States Food and Drug Administration, plant-origin enzyme products are typically sold as nutritional supplements for which drug claims for direct health benefits are disallowed. In that case, label claims are therefore usually vague, and regulations that apply to medications are not relevant; thus, there are no studies required to prove safety or efficacy^(^^2^^)^.

To our knowledge, there are no data available regarding the impact of the use of products containing lipase, protease and amylase, of either plant or animal origin, on nutrient digestibility by healthy dogs. While reports of the efficacy of digestive enzyme therapy in dogs with EPI have been published, the control groups in these studies have consisted of dogs with uncontrolled EPI rather than dogs with normal exocrine pancreatic function^(^^3^^–^^7^^)^. Further, the effect of oral enzyme supplements on serum concentrations of trypsin-like immunoreactivity (TLI) has not been documented. This test is used to diagnose EPI in animals with compatible clinical signs, and is sensitive and specific for exocrine pancreatic function^(^^8^^)^. Thus, it is important to establish whether there is an effect of these products on serum TLI concentrations to clarify interpretation of the test results in patients receiving these supplements.

The objective of the study is to measure macronutrient and energy digestibility by healthy dogs fed a commercial dry canine diet with or without supplementation with exogenous digestive enzymes (of both plant and animal origin) at the dosage recommended by their respective manufacturers, and to determine the effect of enzyme supplementation on serum TLI concentrations. We hypothesise that there will be no effect of exogenous enzyme supplementation on macronutrient and energy digestibility or TLI values.

## Materials and methods

### Animals and design

The study was approved by the Universitat Autonoma de Barcelona ethics committee (CEEAH 1467).

A total of fourteen healthy neutered adult dogs (eight males and six females; median age 8 years, range 4–11 years, weight 13·2 (sd 2·92) kg) were used for this study, divided into two groups of seven dogs according to their sex and body weight. The dogs were kept at the experimental kennels of the Facultat de Veterinaria, Universitat Autònoma de Barcelona and underwent veterinary examination before and after the trial.

The dogs were housed individually in protected covered runs with free access to clean and fresh water; their energy requirements are known from previous trials and food was supplied in adequate amounts to satisfy these requirements and maintain a stable body weight. Three 10-d experimental periods were carried out. In the first period a basal digestibility trial of the commercial dry maintenance canine diet (Nestlé Purina Pro Plan Medium Adult Chicken and Rice, Canine, dry; Nestlé Purina) without enzyme supplement added was conducted including all dogs. In periods 2 and 3, dogs were divided into two groups and received the plant-origin enzyme supplement (product A (Prozyme All-Natural Enzyme Supplement Original Formula for Dogs and Cats; PBI/Gordon Corporation); α-amylase from *Aspergillus oryzae* 2000 SKB/g, cellulase from *A. niger* 50 CU/g, lipase from *A. niger* 30 FIP/g, bromelain from pineapple stem and fruit 8 GDU/g) or the animal-origin enzyme supplement (product B (Pancrezyme, Virbac Animal Health); lipase from porcine pancreas 71 400 USP units/2·8 g, protease from porcine pancreas 388 000 USP units/2·8 g, amylase from porcine pancreas 460 000 USP units/2·8 g) at the doses recommended by the manufacturer (1/2 teaspoon (2·5 g) per cup of food and 1 teaspoon (2·8 g) per meal for products A and B, respectively). In period 2, one group of dogs received product A and the other group received product B and in period 3 the treatments were switched. At the beginning of the study and at the end of each digestibility period, dogs were weighed and fasted for 12 h and blood was collected, processed and submitted to a commercial laboratory (IDEXX Laboratorios, Barcelona, Spain) for analysis of serum TLI.

### Digestibility trial protocol

The digestibility protocol is adapted from the official method of the Association of American Feed Control Officials (AAFCO) Dog and Cat Food Metabolizable Energy Protocols^(^^9^^)^. Each 10-d digestibility trial included 5 d for adaptation and 5 d for total collection of faeces. Daily food intake was recorded. The same batch of diet and enzymes was used for all trials. The dogs were fed once daily at the same time of day. During the collection period, the faeces were collected twice daily, weighed and frozen. After the 5-d collection period, the faeces were weighed again and dried in an oven at 50–60°C until constant weight was reached (3–5 d) to determine DM. After drying, the faeces were ground and mixed and a representative sample of each was taken and frozen at −20°C until analysis. A representative sample of the basal diet was collected on days 1, 5 and 10 of each trial and ground and stored at 5°C prior to analysis.

Apparent total tract digestibility coefficients for DM, organic matter, ether extract (EE; crude fat), crude protein (CP) and gross energy (GE) were calculated as follows:

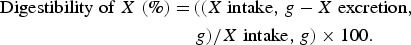


### Sample analysis

The chemical composition of the diet was determined according to the following methods of the AOAC^(^^10^^)^: DM (934.01), ash (942.05), CP (988.05), crude fibre (950.02) and hydrolysed EE (920.39). Hydrolysed EE, DM and CP were also analysed in the faeces. GE was determined in food and excreta using an adiabatic bomb calorimeter (IKA-Kalorimeter system C4000; Janke-Kunkel). To calculate digestible energy, the GE digestibility percentage was multiplied by the GE of the food. The metabolisable energy (ME) of the experimental diet was calculated from the digestible energy and the CP content of the diet according to the National Research Council^(^^11^^)^ proposed equation: ME (kcal/g) = DE – (1·04 × g CP).

### Statistical analysis

The number of animals per group was chosen according to AAFCO Dog and Cat Food Metabolizable Energy Protocols^(^^9^^)^, which require at least six dogs to perform a digestibility test.

Statistical analysis was done using SAS 9.3 (SAS Institute, Inc.). Digestibility values and serum TLI were compared for basal diets and product A *v*. B using repeated measures. The model included treatment as a fixed effect and dog as a random effect. A model including period as a fixed effect and dog as a random effect was also used to analyse the effect of period. Data are presented as means with their standard errors unless otherwise stated. The α level of significance was set at 0·05. Mean separation for multiple comparisons was done using Tukey's correction.

## Results

The dogs maintained stable body weights throughout the experiment (13·4 (se 2·97) kg). The chemical composition and energy content for the basal diet (averaged from three samples) are presented in Table 1. Total tract apparent digestibility coefficients of DM, organic matter, CP, EE and GE are presented in Table 2. There were no differences in macronutrient and energy digestibility between enzyme treatments. When comparing enzyme-supplemented *v*. basal diet digestibility coefficients, EE (crude fat) digestibility was higher for the basal diet compared with the animal-origin enzyme treatment. Serum TLI was not affected by supplementation with either enzyme product (25·1, 23·3 and 24·8 µg/l for basal diet and products A and B, respectively, *P* = 0·682) and was within normal reference ranges (2·5–50 µg/l) at all times.

**Table 1.** Chemical composition (fresh matter basis) of the basal diet (average of three measurements)

Nutrient	Concentration (g/100 g)
DM	92·2
Crude protein	25·8
Ether extract/crude fat	15·8
Crude fibre	2·9
Ash	7·5
Gross energy (MJ/kg)	19·8
Metabolisable energy (MJ/kg)*	15·7

* Calculated using gross energy, energy digestibility and crude protein of the diet according to the National Research Council^(^^11^^)^.

**Table 2.** Digestibility (expressed as percentage) of energy and macronutrients of dogs (*n* 14) fed a basal diet supplemented with a plant- or animal-origin enzyme supplement* (Mean values with pooled standard errors)

	Basal	Plant-origin enzyme	Animal-origin enzyme	se	*P* (treatment)
DM	79·4	79·3	79·6	0·53	0·880
Organic matter	84·1	83·4	83·6	0·40	0·289
Crude protein	79·8	78·1	79·1	0·52	0·075
Crude fat	94·7^a^	93·9^a,b^	93·5^b^	0·24	0·006
Gross energy	84·8	84·3	84·4	0·36	0·364

^a,b^ Mean values within a row with unlike superscript letters were significantly different (α = 0·05).

* Mean separation was done using Tukey's correction.

## Discussion

This study is the first to report comparisons of the digestibility coefficients of energy and macronutrients of a maintenance diet with or without supplementation with two different enzyme products (one plant and one animal origin) by adult healthy dogs.

Data regarding exogenous digestive enzyme supplementation in healthy dogs are scarce, especially compared with other species such as pigs and poultry, where their use is routine^(^^12^^)^. Most of the published studies in dogs have assessed the use of carbohydrases (rather than mixtures of amylase, protease and lipase) when using ingredients with antinutritional factors and high fibre content, in order to improve nutrient availability. The authors of one study compared diets based on rice, sorghum and maize with or without an enzyme mixture at 1 ml per ton (including xylanase, α-amylase, β-glucanase, hemicellulase, pectinase and endoglucanase) in dogs and showed no impact of enzyme treatment on digestibility of protein, fat and energy^(^^13^^)^. Similarly, Pacheco *et al*.^(^^14^^)^, when comparing diets with different amounts of full-fat rice bran, found that the inclusion of a mixture of carbohydrases, phytase and protease (0·4 and 0·8 g/kg of diet) did not affect digestibility values. Sá *et al*.^(^^15^^)^ added a mixture of carbohydrases and phytase to canine diets including wheat bran, pre- and post-extrusion, and found no significant effect at the inclusion levels used. Another group reported no effects of exogenous protease and cellulose on canine digestibility of diets based on poultry meal *v*. soyabean meal^(^^16^^)^; however, Félix *et al*.^(^^17^^)^, comparing diets also based on poultry and soyabean meals, found that the inclusion of mannanase at 0·01 % resulted in improved macronutrient digestibility. Overall, digestive enzyme supplementation (mainly carbohydrases) in feed does not seem to have a marked effect on canine nutrient and energy digestibility.

This is similar to our findings, although our study used commercial products providing mixtures of α-amylase, cellulase, lipase and bromelain (plant-origin product) or lipase, protease and amylase (animal-origin product). The dosages used were those recommended by the manufacturer: half a teaspoon (2·5 g) per cup for senior dogs (they recommend a quarter teaspoon for young adults) for the plant-origin product and one teaspoon (2·8 g) per meal for the animal-based one, without incubation time (as per instructions of the manufacturer). The only effect documented was a slightly higher crude fat digestibility for the basal diet compared with the animal-origin enzyme treatment, which is probably a period effect due to slightly lower values in period 2, and not biologically significant (94·7 *v*. 93·5 %). This result was expected, since the basal diet used is comparable with many maintenance canine diets in its formulation and processing which help ensure adequate digestibility and bioavailability of nutrients.

Improved digestibility with the use of animal-based digestive enzymes in dogs with EPI, in comparison with untreated dogs with EPI, has been documented^(^^3^^–^^7^^)^; however, our results do not support their efficacy in healthy pets at the recommended dosages. It is unknown if higher dosages or a different protocol (i.e. pre-incubation) would have resulted in a positive effect.

Our study is the first to assess the effect of exogenous digestive enzyme supplementation on serum TLI in dogs. At the dosages recommended by the manufacturer, the inclusion of either plant- or animal-origin enzyme supplements did not affect TLI values, which remained normal throughout the study. Veterinarians might prescribe these enzymes before any testing is performed (especially in dogs with severe disease) and some pet owners may pre-emptively utilise them for their pets with non-specific diarrhoea. Our results show that TLI is unaffected by enzyme supplementation at the recommended dose; thus, this test can be reliable in patients receiving these supplements, especially if they do not have EPI. The effect of enzyme supplementation in TLI of dogs that do have EPI is still unknown.

In conclusion, the supplementation of a maintenance canine dry diet with recommended doses of exogenous digestive enzymes, plant or animal origin, does not result in improvements of digestibility of protein, fat or energy in healthy adult dogs and does not affect serum TLI concentrations in these individuals. Thus, their routine use in healthy pets is not recommended.
